# Sepsis in critically ill dogs: identification of clinical and laboratory risk factors and evaluation of sepsis scoring systems

**DOI:** 10.1093/jvimsj/aalag168

**Published:** 2026-08-13

**Authors:** Donatienne Castelain, Mathilde Laetitia Pas, Marcin Skotarek, Filip Boyen, Bart Pardon, Dominique Paepe

**Affiliations:** Department of Internal Medicine, Reproduction and Population Medicine, Faculty of Veterinary Medicine, Ghent University, Salisburylaan 133, Merelbeke, 9820, Belgium; Department of Internal Medicine, Reproduction and Population Medicine, Faculty of Veterinary Medicine, Ghent University, Salisburylaan 133, Merelbeke, 9820, Belgium; Small Animal Department, Faculty of Veterinary Medicine, Ghent University, Salisburylaan 133, Merelbeke, 9820, Belgium; Department of Pathobiology, Pharmacology and Zoological Medicine, Faculty of Veterinary Medicine, Ghent University, Salisburylaan 133, Merelbeke, 9820, Belgium; Department of Internal Medicine, Reproduction and Population Medicine, Faculty of Veterinary Medicine, Ghent University, Salisburylaan 133, Merelbeke, 9820, Belgium; Small Animal Department, Faculty of Veterinary Medicine, Ghent University, Salisburylaan 133, Merelbeke, 9820, Belgium

**Keywords:** antimicrobial stewardship, blood culture, logistic regression, prognosis, septicemia

## Abstract

**Background:**

Being a life-threatening syndrome, rapid detection of sepsis in critically ill dogs is mandatory.

**Hypothesis/Objectives:**

Determine risk factors associated with sepsis in critically ill dogs and evaluate diseases and scoring systems associated with sepsis or mortality.

**Animals:**

One hundred thirty critically ill dogs.

**Methods:**

Prospective single-center cohort study. Logistic regression and classification and regression tree (CART) analysis were performed to assess clinical and laboratory variables as risk factors for sepsis. Diagnostic and prognostic performance of available scoring systems was evaluated.

**Results:**

Sepsis was confirmed in 29 of 130 critically ill dogs (22.3%), and 18 died, with a short-term mortality rate of 62.1%. Pneumonia (odds ratio [OR] = 4.8; 95% confidence interval [CI] = 1.8-13.1; *P* = .002) and acute kidney injury (AKI; OR = 7.4; 95% CI = 2.2-25.1; *P* = .001) were associated with sepsis. Multivariable logistic regression identified dyspnea (OR = 6.5; 95% CI = 1.6-26.0; *P* = .01), respiratory rate (≥38 per minute; OR = 3.9; 95% CI = 1.1-13.1; *P* = .03), and alanine aminotransferase activity (≥109.5 U/L; OR = 3.8; 95% CI = 1.1-12.8; *P* = .03) as risk factors for sepsis. When combined, these factors yielded a sensitivity of 35.0% and specificity of 92.8%. The CART analysis identified body weight, tachypnea, hypotension, hyperchloremia, hyperlactatemia, and hyponatremia as risk factors for sepsis (sensitivity, 58.6%; specificity, 97.0%). The APPLE FAST score (OR = 2.9; 95% CI = 1.2-7.1; *P* = .02) was associated with sepsis. The APPLE FAST score (OR = 3.2; 95% CI = 1.4-7.2; *P* = .01) was the strongest predictor of mortality.

**Conclusions and clinical importance:**

Pneumonia and AKI were associated with sepsis. Identified risk factors may guide veterinarians in the rapid recognition of sepsis. The APPLE FAST score was associated with sepsis and mortality and may support diagnostic and therapeutic decision-making for critically ill dogs with sepsis.

## Introduction

Sepsis, a life-threatening organ dysfunction syndrome caused by a dysregulated host response to infection, is a leading cause of mortality in humans and animals.^1^ Sepsis is commonly observed in critically ill dogs, with studies reporting an incidence from 5.0% to 10.0% in referral hospitals.^2^^,^^3^ As mortality rates increase to 80.0%, delays in diagnosis and therapeutic interventions are associated with poor outcomes in dogs, emphasizing the importance of prompt initiation of IV, broad-spectrum, and bactericidal antimicrobial treatment, along with supportive treatment.^1^^,^^3–5^ Although data on hospitalization times and costs in dogs with sepsis are limited, human medical literature suggests a substantial burden.^4^^,^^6^ In human medicine, definitions of sepsis have evolved considerably, but a universally accepted definition is currently lacking in veterinary medicine.^1^^,^^3^ Despite Sepsis-3 criteria being adopted in human medicine, small animal veterinarians continue to rely on the systemic inflammatory response syndrome (SIRS) criteria combined with infection to define sepsis.^3^ The original SIRS criteria^7^—respiratory rate (RR) >20 per minute (excluding panting), heart rate >120 beats per minute (bpm), temperature <38.1 or >39.2 °C, and leukocyte count <6.0 or >16.6 × 10^9^/L, demonstrate high sensitivity but limited specificity in identifying septic patients, limiting the utility as a sole diagnostic tool for sepsis in dogs.^1^^,^^6^^,^^8^ Modified SIRS criteria have been explored. However, no consensus has been reached regarding their optimal use.^1^^,^^7^^,^^9^ A veterinary consensus panel is engaged in efforts to establish a globally applicable definition of sepsis in dogs.^1^ Diagnosis of sepsis is complicated by the nonspecific nature of clinical signs and absence of a definitive reference standard to confirm bacteremia. Blood culture results may be subject to false-positive or false-negative outcomes, limiting their accuracy as a diagnostic tool.^1^^,^^10^ In humans, alternative tools such as the quick sequential organ failure assessment (qSOFA) score were developed with sensitivities and specificities reported between 46.0%-75.2% and 38.5%-82.9%, respectively.^6^ The acute patient physiologic and laboratory evaluation (APPLE) FAST score and 2 modified versions of the original SIRS criteria (hereafter referred to as “modified SIRS2 criteria” and “modified SIRS3 criteria”) were originally established for dogs.^7^^,^^11^ However, their clinical applicability is limited because of lack of research on their performance and validation.^5^^,^^7^^,^^8^^,^^11^^,^^12^ No studies have evaluated both human and dog-derived scoring systems for predicting sepsis or mortality in critically ill dogs. Additionally, to our knowledge, risk factors associated with sepsis in this population remain unexplored. Identification of risk factors could improve diagnostic accuracy and clinical decision-making, resulting in a more rational use of critically important antimicrobials. Furthermore, these risk factors could guide both veterinary practitioners and dog owners in the management of critically ill dogs and may provide added value when integrated into new clinical scoring systems.

Therefore, our objectives were to: (1) identify the most prevalent diseases and comorbidities associated with sepsis or mortality in critically ill dogs; (2) determine clinical and laboratory variables associated with sepsis in critically ill dogs that are positive for SIRS criteria by logistic regression and classification and regression tree (CART) analysis; and (3) determine the association of existing sepsis and critical illness scoring systems from human and canine medicine with sepsis or mortality in critically ill dogs.

## Materials and methods

### Study cohort

A prospective, exploratory study was conducted at the Small Animal Veterinary Hospital of Ghent University, after approval from the Ethical Committee (EC 2022-049; approval date September 27, 2022). This study adhered to the strengthening the reporting of observational studies in epidemiology (STROBE) guidelines.^13^ The study population consisted of 2 groups: critically ill non-septic and critically ill septic dogs. Owner consent forms were signed before participation. Critically ill non-septic dogs were defined as those admitted to the emergency department or intensive care unit, presenting with severe and potentially life-threatening conditions and meeting 2 or more SIRS criteria. The SIRS criteria were assessed based on clinical variables, as recommended previously,^14^ and laboratory reference values of the laboratory machines used (see “Sam*ple collection–Sampling*”): tachypnea (RR >40 per minute), tachycardia (heart rate >140 bpm), hypo- or hyper-thermia (<37.2 or >39.2 °C), leukocytosis (>16.760/μL), and left shift leukocytosis (band neutrophils >5.0%).^14^^,^^15^ Without intervention, this group of dogs is expected to die immediately or within hours (subjectively, based on clinical judgment).

Dogs of the sepsis group were required to meet the inclusion criteria for being critically ill, fulfill the SIRS criteria (described above), and either have confirmed sepsis or be strongly suspected of having sepsis. Confirmed sepsis was defined as a dog having a positive blood culture with evidence of organ dysfunction remote from the site of infection or a dog that had more than one focus of infection (blood culture not included, 2 different organs) from which the same pathogen was isolated with evidence of organ dysfunction remote from the site of infection. Organ dysfunction was defined as the presence of one or more of the following: circulatory abnormalities (hypotension; systolic blood pressure [SBP] <90 mm Hg in combination with hypoperfusion [lactate >2.5 mmol/L]), pulmonary abnormalities (tachypnea [RR >40 per minute, not attributed to panting] combined with dyspnea), renal dysfunction (evidence of grade II or higher acute kidney injury [AKI] according to International Renal Interest Society [IRIS guidelines]^16^), thrombocytopenia <148,000/μL, hepatic dysfunction (total bilirubin concentration >15.0 μmol/L), with or without abnormalities of the central nervous system (Glasgow coma scale <18). Strongly suspected sepsis was defined as dogs having all of the following criteria: (1) negative blood culture (or not performed), but confirmed infection elsewhere in the body (eg, enteritis, pneumonia, septic peritonitis, pyometra, urinary tract infection); (2) evidence of organ dysfunction remote from the site of infection, as defined above; and (3) at least 2 of the following abnormalities: septic shock (need for vasopressors despite adequate fluid resuscitation to maintain SBP >90 mm Hg), fever (>39.2 °C), hypothermia (<37.2 °C), hypotension (SBP <90 mm Hg), accelerated capillary refill time (CRT, <1 second), neutropenia (<2950/μL), left shift leukocytosis (band neutrophils >5.0%), leukopenia (<5050/μL), toxic changes in the neutrophils (>5.0%), or hypoglycemia (<4.1 mmol/L).^14^^,^^15^ These criteria were selected based on the Sepsis-3 definition used in humans, supported by the available veterinary and human medical literature, and further refined by expert opinion.^1^^,^^6^^,^^17^

Acute kidney injury grade II or higher was defined based on the IRIS criteria^16^ as follows: dogs presenting with acute (<14 days) signs were included if they met 1 of the 2 conditions:

1) Mild azotemia defined as serum creatinine concentration >159.0 μmol/L and blood urea nitrogen concentration >9.6 mmol/L (upper reference limit, in-house IDEXX Catalyst Dx chemistry analyzer [IDEXX Laboratories Inc, Westbrook, Maine]), persisting at least 24 hours after correction of dehydration and other prerenal factors, combined with one of the following characteristics of AKI:

- Medical imaging findings compatible with AKI,
- Glucosuria without supplementation and with normal blood glucose concentration (<10.0 mmol/L),
- Urine protein/creatinine ratio (UPC) >0.5 (proteinuria) with non-active sediment,
- Evidence of acute tubular injury on urinalysis,
- Dogs for which oliguria or azotemia or both were fluid volume-responsive (fluid responsiveness defined as an increase in urine production to >1.0 mL/kg/h within 6 hours, or a decrease in serum creatinine concentration or both to baseline over 48 hours).

2) A (non-azotemic) increase from baseline serum creatinine concentration by >26.4 μmol/L (>0.3 mg/dL) within a 48-hour interval after correction of dehydration.

Dogs with findings of chronic kidney disease (eg, unclear corticomedullary distinction, hyperechogenic cortex) on ultrasonography or receiving medication influencing renal perfusion (eg, angiotensin-converting enzyme inhibitors, angiotensin receptor blockers, dopamine, calcium channel blockers) were excluded from AKI group. Exclusion criteria for all dogs were severe anemia (hematocrit <14.0%), severe thrombocytopenia (<20,000/μL), body weight <2.0 kg, or dogs in cardiogenic or hypoxemic shock, because of ethical and practical considerations associated with the additional blood sampling for the study.

### Physical and blood examination

Signalment (breed, age, sex) and body weight (kg) were determined. History consisted of questions on the medical condition, and additionally, information on the administration of antimicrobials (type, dose, duration, and frequency of administration) and IV fluids before admission or blood culture sampling. Subsequently, 26 clinical risk factors were comprehensively evaluated: mental status (alert, lethargy, apathy, comatose), RR (per minute), breathing type (normal, dyspnea), lung auscultation (normal, wheezes, crackles, muffled, increased lung sounds), heart rate (bpm), heart auscultation (normal, arrhythmia, heart murmur, muffled), mucosal membranes (pink, light pink, pale, hyperemic, cyanotic, icteric, petechiae), CRT (seconds), lymph nodes (normal, locally enlarged, generally enlarged), pulse quality (normal, weak, strong), temperature (°C), skin turgor (seconds), body condition score (on a 9-point scale and as underweight [1-3], ideal weight [4-6], overweight [7-9]),^18^^,^^19^ inspection of the abdomen (normal, distended), abdominal palpation (normal, tense [surface or deep]), abdominal undulation (free fluid; negative, positive), nervous system (alert, lethargic, stuporous, comatose), Glasgow coma scale, decubitus (normal, lateral, sternal), lameness (caused by injury), joints (normal, swollen [monoarticular or polyarticular]), vaginal discharge (absent, serous, hemorrhagic, purulent), wounds, urination abnormalities (normal, polyuria, oliguria, anuria, hematuria, pollakiuria, dysuria, incontinence), dental health (normal, limited plaque without gingivitis, plaque with gingivitis, periodontitis with gum recession and exposed tooth roots), and SBP (mm Hg). Non-invasive SBP measurement was conducted using Doppler ultrasonic technique. Point-of-care ultrasonography (POCUS) of the thorax and abdomen was performed to screen for abdominal, pleural, or pericardial effusion and conditions that warranted urgent intervention. After stabilization, abdominal ultrasonography, thoracic radiography, and other diagnostic tests were performed based on the clinician’s discretion.

### Sample collection—sampling

After the physical examination and before initiating fluid therapy, blood was collected from the jugular vein using a 21-gauge (G) needle and a 10-20 mL syringe and subsequently divided to collect EDTA plasma, serum, and heparinized plasma samples. Hematology, biochemistry, electrolytes (sodium [Na^+^], potassium [K^+^], and chloride [Cl^-^]), total bilirubin, and lactate were determined using the IDEXX Procyte Dx analyzer (IDEXX Laboratories Inc., Westbrook, Maine) and IDEXX Catalyst Dx chemistry analyzer (IDEXX Laboratories Inc., Westbrook, Maine). Lactate was analyzed immediately after blood sampling to ensure accurate results. At the same time as blood sampling, blood cultures were collected aseptically as described below. Blood smears were prepared using the wedge technique and immediately stained with Diff-Quik (Diff-Quik Fix, Median Diagnostics, Düdingen, Switzerland).^20^ The smears were stored at room temperature in a light-protected environment. Evaluation was performed by a veterinarian in collaboration with an European Board of Veterinary Specialists in Small Animal Internal Medicine to determine the percentage of toxic neutrophils and band neutrophils, and the severity of thrombocytopenia. The number of thrombocytes was estimated based on examination of multiple microscopic fields, considering the number of platelets per high-power field (hpf, thrombocytopenia indicated as <10-15/hpf), and the presence of platelet aggregates and macrothrombocytes. After centrifugation, the remaining serum and plasma were stored at -80°C in aliquots of 250-300 μL within 24 hours after collection.

### Sample collection—blood culture processing

Blood culture was collected after aseptically preparing (clipping, scrubbing with chlorhexidine 4%, and disinfection with 70% isopropyl alcohol) the designated area. After air drying the skin, blood was taken with a 21G needle and a 10-20 mL syringe. After disinfection of the stopper with 70% isopropyl alcohol, the collected blood was injected into the blood culture bottle, using a second needle (2-needle technique).^21^ Aerobic enriched media (BACTEC™ Plus Aerobic medium 8-10 mL or BD BACTEC™ Peds Plus™ medium 1-3 mL), anaerobic medium (BACTEC™ Plus Anaerobic medium 8-10 mL; BD, Erembodegem, Belgium) or both were used. The volume of the collected blood was determined based on the dog’s body size and clinical condition. Subsequently, the blood culture bottles were incubated in an automated machine for the detection of microbial growth at 35 °C (BACTEC™ FX, BD, Erembodegem, Belgium). If no microbial growth was detected after 5 days of incubation, the samples were considered negative. If bacterial growth was present, samples underwent microbial identification and antimicrobial susceptibility testing at an external laboratory as well as at the Faculty of Veterinary Medicine Department of Pathobiology, Pharmacology and Zoological Medicine using matrix-assisted laser desorption/ionization time-of-flight mass spectrometry (MALDI-TOF MS).

### Sepsis and critical illness scoring systems

Different existing scoring systems, developed either for sepsis diagnosis or prognosis, were evaluated for their association with sepsis or mortality. Diagnostic scores included: the original SIRS, modified SIRS2, and modified SIRS3, whereas prognostic scores consisted of the qSOFA, the APPLE FAST score, Lactate 2 (L2) qSOFA, L3qSOFA, L4qSOFA, and L5qSOFA. An overview of the evaluated scoring systems is presented in Supplementary Table 1. Supplementary Figure 1 shows the criteria used for the APPLE FAST score.^11^

The diagnostic criteria for the original SIRS, as proposed previously, included the following variables: RR >20 per minute (excluding panting), heart rate >120 bpm, temperature <38.1 or >39.2 °C, and leukocyte count <6.0 or >16.6 × 10^9^/L.^7^ A diagnosis of SIRS was confirmed when at least 2 criteria were met. The modified SIRS criteria (modified SIRS2) were similar to the original SIRS criteria, with adjustments made for RR >40 per minute and heart rate >150 bpm.^7^ The modified SIRS3 was fulfilled when 3 modified criteria were fulfilled.^7^ The original qSOFA score is a scoring system consisting of 3 clinical criteria: RR ≥22 per minute (excluding panting), SBP ≤100 mm Hg, and altered mentation. Each criterion was assigned one point when the criterion was met, resulting in a total score ranging from 0 to 3.^5^^,^^12^ In our study, a modified qSOFA score was added by adjusting the RR threshold to >40 per minute, reflecting the normal upper limit in dogs.

L2qSOFA, L3qSOFA, L4qSOFA, and L5qSOFA are modified versions of the original qSOFA score, described previously,^4^ developed to enhance the predictive accuracy for both sepsis and mortality in dogs by incorporating serum lactate concentrations.^4^^,^^5^ In these models, one additional point was assigned to the original qSOFA score if the lactate concentration exceeded specific thresholds. Specifically, one point was added to the L2qSOFA score when lactate concentrations were >2.0 mmol/L; to the L3qSOFA score when lactate was >3.0 mmol/L; to the L4qSOFA score when lactate was >4.0 mmol/L; and to the L5qSOFA score when lactate exceeded 5.0 mmol/L. If lactate concentrations remained below the defined threshold (≤2.0 mmol/L), the original qSOFA score was retained.

The APPLE FAST score included the following variables: blood glucose (mmol/L), serum albumin (g/L), blood lactate concentration (mmol/L), platelet count (10^3^/μL), and a structured mentation score ranging from 0 to 4. The mentation score was defined as follows: 0 = normal mentation; 1 = able to stand unassisted, responsive but dull; 2 = able to stand only with assistance, responsive but dull; 3 = unable to stand, responsive; and 4 = unable to stand, unresponsive. The individual components were weighted, and the cumulative APPLE FAST score ranged from 0 to a maximum of 50, with higher scores indicating higher clinical severity.^11^

### Statistical analysis

Data initially were collected in a Microsoft Excel worksheet (Microsoft Inc., Redmond, Washington) and subsequently transferred to IBM SPSS Statistics for Windows, Version 29.0 (IBM Corp., Armonk, NY) for statistical analysis. First, to determine the association between sepsis or mortality and other diseases, logistic regression was performed. Second, to assess clinical and laboratory variables as risk factors for sepsis, another logistic regression model was applied, with sepsis (0/1) as the outcome variable. Continuous variables were evaluated both continuously and categorically. Categorical variables were derived based on quartiles, deviations from normal reference values, and binary classifications determined by receiver operating characteristic (ROC) curve analysis. Breeds were categorized according to weight classifications: small (<15 kg), medium (15-25 kg), and large (>25 kg).^22^ Pearson and Spearman correlation coefficients were used to evaluate correlations among significant continuous variables. If a correlation coefficient was ≥0.6, only the most significant variable was retained for further modeling. Chi-squared tests were used to assess associations between categorical variables.

In addition to numerical collinearity screening, conceptual collinearity also was evaluated to ensure biological interpretability. Doing so may be particularly relevant for variables that reflect overlapping domains of organ dysfunction (eg, lactate, hypotension, altered mentation). In practice, however, most overlapping predictors already had been removed during univariable screening based on statistical criteria (*P*-value). As a result, conceptual collinearity served primarily as a secondary check because biologically guided alternative models excluding overlapping variables did not yield significant multivariable results.

Results are presented as mean ± SD and median (minimum [min]-maximum [max]). Two multivariable logistic regression models were constructed: the first included only clinical variables, whereas the second incorporated both clinical and laboratory variables. The model-building procedure involved univariable testing of each variable’s association with sepsis. Variables with *P* < .2 in the univariable analysis and more than 90 observations were eligible for the multivariable analysis, which followed a stepwise backward elimination approach, sequentially removing non-significant variables. A significance level of *P* < .05 was used for all statistical tests.

As an alternative approach to identify risk factors associated with sepsis, CART analysis was performed using both categorical variables and continuous variables. A decision tree was constructed by splitting data to maximize homogeneity within nodes and minimize impurity until either homogeneity or predefined stopping criteria in a node were achieved with respect to the value of the objective variable, as measured by the Gini index. The minimum change in improvement was set at 0.0001. The growth limit was defined as 5 observations in the parent node and 2 in the child node, whereby the maximum tree depth was determined at 4. The number of surrogates was set to automatic, enhancing model robustness and predictive accuracy. Diagnostic performance of the decision tree was evaluated using diagnostic accuracy, sensitivity, and specificity. Because of the limited sample size, cross-validation was used for validation.

Third, to evaluate the association between scoring systems with sepsis or mortality, logistic regression analysis was performed. Additionally, the diagnostic accuracy, sensitivity, and specificity of the various scoring systems were calculated from a 2 × 2 table.

## Results

### Diseases and their association with sepsis

A total of 130 critically ill dogs were included, of which 22.3% (29/130) were diagnosed with sepsis using the new sepsis definition developed for our study. Demographic variables are summarized in Table 1. In Table 2, common diseases affecting critically ill dogs are presented, as well as their association with sepsis or mortality. Diseases may have coexisted as comorbid conditions (eg, sepsis combined with pneumonia). Sepsis, enteritis, pancreatitis, pneumonia, septic peritonitis, AKI, and acute hemorrhagic diarrhea syndrome were most prevalent. Overall mortality rate (during or shortly after hospitalization) among critically ill dogs was 36.9% (48/130). Mortality rate for dogs diagnosed with sepsis was 62.1% (18/29). Septic dogs had an increased risk of death (odds ratio [OR] = 3.9; 95% confidence interval [CI] = 1.6-9.2; *P* = .002). For dogs with sepsis, mean ± SD and median time between inclusion and death were 4.1 ± 3.1 days and 3.0 days (min-max, 1-13 days). For dogs without sepsis, mean ± SD and median time between inclusion and death were 4.0 ± 3.1 days and 3.0 days (min-max, 1-15 days).

**Table 1:** Demographic variables of the study population per group of dogs.

**Variable**	**Group**	**Mean**	**±SD**	**Median (min.-max.)**	**% (*n*/*N*)**
**Age upon admission (years)**	All critically ill dogs	6.1	3.7	6.0 (.3-15.0)	/
	Dogs with sepsis	6.3	3.6	6.0 (.3-12.3)	/
	Dogs without sepsis	6.0	3.7	6.0 (.5-15.0)	/
**Body weight upon admission (kg)**	All critically ill dogs	23.5	13.4	23.0 (2.5-71.0)	/
	Dogs with sepsis	23.9	13.5	23.9 (4.6-71.0)	/
	Dogs without sepsis	23.9	13.6	23.0 (2.5-62.5)	/
**Sex**	Male	/	/	/	54.6% (71/130)
	Female	/	/	/	45.4 (59/130)

Abbreviations: min. = minimum; max. = maximum; n = number of male/female dogs; N = number of dogs; SD = standard deviation.

**Table 2:** Common diseases in 130 critically ill dogs and their association with sepsis and mortality.

**Diseases **	**Disease% (*N*)**	**Sepsis**			**Mortality**		
		**Septic dogs per disease% (*N*)**	***P*-value**	**OR (95% CI)**	**Mortality per disease% (*N*)**	***P*-value**	**OR (95% CI)**
**Sepsis**	22.3 (29)	100 (29)			62.1 (18)	**.002**	3.9 (1.6-9.2)
**Enteritis**	18.5 (24)	12.5 (3)	.21	.44 (.1-1.6)	29.2 (7)	.4	.7 (.2-1.7)
**Pancreatitis**	18.5 (24)	16.7 (4)	.5	.6 (.2-2.1)	45.8 (11)	.3	1.6 (.6-3.9)
**Pneumonia**	15.4 (20)	50.0 (10)	**.002**	4.8 (1.8-13.1)	55.0 (11)	.07	2.4 (.9-6.3)
**(Septic) peritonitis**	13.1 (17)	29.4 (5)	.5	1.5 (.5-4.8)	52.9 (9)	.1	2.5 (.9-7.2)
**AKI (≥ grade II)**	10.0 (13)	61.5 (8)	**.001**	7.4 (2.2-25.1)	69.2 (9)	**.01**	5.0 (1.4-17.4)
**AHDS**	9.2 (12)	16.7 (2)	.6	.7 (.1-3.3)	16.7 (2)	.1	.3 (.07-1.5)
**Abscess**	9.2 (12)	16.7 (2)	.6	.7 (.1-3.3)	25.0 (3)	.4	.5 (.1-2.1)
**Bacterial cystitis**	7.7 (10)	40.0 (4)	.2	2.5 (.7-9.7)	20.0 (2)	1.0	1.0 (.07-14.6)
**FUO**	7.7 (10)	.0 (0)	1.0	/	.0 (0)	1.0	/
**Neoplasia**	7.7 (10)	30.0 (3)	.5	1.5 (.4-6.4)	70.0 (7)	**.04**	4.5 (1.1-18.3)
**Endocarditis**	4.6 (6)	33.3 (2)	.5	1.8 (.3-10.3)	66.7 (4)	.1	3.6 (.6-20.7)
**DKA**	4.6 (6)	16.7 (1)	.7	.7 (.1-6.1)	50.0 (3)	.5	1.8 (.3-9.1)
**Pyometra**	3.8 (5)	40.0 (2)	.3	2.4 (.4-15.2)	20.0 (1)	.4	.4 (.05-3.8)
**Prostatitis**	3.8 (5)	.0 (0)	1.0	/	20.0 (1)	.4	.4 (.05-3.8)
**DIC**	3.1 (4)	25.0 (1)	.9	1.2 (.1-11.7)	75.0 (3)	.1	5.4 (.5-53.4)
**(Poly)arthritis**	3.1 (4)	25.0 (1)	.9	1.2 (.1-11.7)	.0 (0)	1.0	/
**Pyothorax**	2.3 (3)	33.3 (1)	.6	1.8 (.2-20.2)	33.3 (1)	.9	.9 (.08-9.6)
**Trauma**	2.3 (3)	100 (3)	1.0	/	66.6 (2)	.3	3.5 (.3-39.9)
**Intoxication**	.7 (1)	100 (1)	1.0	/	100 (1)	1.0	/
**GDV**	.7 (1)	.0 (0)	1.0	/	100 (1)	1.0	/

Boldface indicates statistically significant results (*P* < .05). Abbreviations: AHDS = acute hemorrhagic diarrhea syndrome; AKI = acute kidney injury; DIC = disseminated intravascular coagulation; DKA = diabetic ketoacidosis; FUO = fever of unknown origin; GDV = gastric dilatation volvulus; N = number of dogs; OR = odds ratio; 95% CI = 95% confidence interval.

### Risk factors associated with sepsis

Results of univariable analysis, emphasizing significant clinical and laboratory variables, are presented in Table 3. Non-significant variables are provided in Supplementary Table 2. Although RR as a continuous variable was significantly associated with sepsis (*P* = .01; Supplementary Table 2), the OR of 1.0 (95% CI = 1.0-1.1) indicates a clinically negligible effect. A similar pattern was observed for sodium, with a 95% CI including 1.0.

**Table 3:** Significant results of univariable logistic regression analysis for the association of clinical and laboratory variables with sepsis in 130 critically ill dogs.

**Variable **	**Categories**	**Septic dogs% (number of septic dogs/number of dogs in the category)**	**Non-septic CI dogs% (number of non-septic dogs/number of dogs in the category)**	***P*-value**	**OR**	**95% CI**	
**Clinical variables**							
Respiratory rate (per minute) (excluding panting in *n* = 42)	<38 (ref) ≥38	10.9 (5/46) 34.8 (16/46)	89.1 (41/46) 65.2 (30/46)	.009	4.4	1.4	13.3
Dyspnea	Absent (ref) Present	19.1 (22/115) 50.0 (7/14)	80.9 (93/115) 50.0 (7/14)	.01	4.2	1.3	13.3
Heart rate (beats per minute)	≤104 (ref) 104 < HR < 128 128 < HR < 155 155 < HR < 211	34.8 (8/23)	65.2 (15/23)	.09			
		6.1 (2/33)	93.9 (31/33)	.01	.1	.02	.6
		27.3 (9/33)	72.7 (24/33)	.5	.7	.2	2.2
		22.5 (9/40)	77.5 (31/40)	.3	.5	.2	1.7
Heart auscultation	Normal (ref) Arrhythmia Heart murmur Muffled	16.8 (16/95) 66.6 (2/3) 28.6 (8/28) 75.0 (3/4)	83.2 (79/95) 33.3 (1/3) 71.4 (20/28) 25.0 (1/4)	.03 .07 .2 .02	9.9 2.0 14.8	.8 .7 1.4	115.6 5.3 151.6
Nervous system	Alert (ref) lethargic Stuporous Coma	15.0 (15/100) 47.6 (10/21) 66.6 (2/3) 50.0 (1/2)	85.0 (85/100) 52.4 (11/21) 33.3 (1/3) 50.0 (1/2)	.004 .002 .05 .2	5.2 11.3 5.7	1.9 1.0 .3	14.2 133.0 95.6
Glasgow coma scale	>18 (ref) <18	14.1 (9/64) 55.0 (11/20)	85.9 (55/64) 45.0 (9/20)	<.001	7.5	2.4	23.1
Recumbency	Normal (ref) Lateral/sternal	15.1 (11/73) 30.8 (16/52)	84.9 (62/73) 69.2 (36/52)	.04	2.5	1.1	6.0
Recumbency	Normal (ref) Sternal Lateral	15.1 (11/73) 14.3 (3/21) 41.9 (13/31)	84.9 (62/73) 85.7 (18/21) 58.1 (18/31)	.01 .9 .004	.9 4.1	.2 1.6	3.7 10.6
**Complete blood count**							
Lymphocytes (10^9^/L)	<3.1 (ref) ≥3.1	17.5 (17/97) 40.0 (12/30)	82.5 (80/97) 60.0 (18/30)	.01	3.1	1.3	7.7
Monocytes (10^9^/L)	<5.7 (ref) ≥5.7	18.1 (21/116) 72.7 (8/11)	81.9 (95/116) 27.3 (3/11)	<.001	12.1	2.9	49.3
**Biochemistry**							
Albumin/globulin (A/G)	≤.7 (ref) .7 < A/G < 0.8 .8 < A/G < 1.0 1.0 < A/G < 1.7	34.7 (17/49) 10.7 (3/28) 15.6 (5/32) 14.3 (2/14)	65.3 (32/49) 89.3 (25/28) 84.4 (27/32) 85.7 (12/14)	.06 .03 .07 .2	.2 .3 .3	.06 .1 .06	.9 1.1 1.6
Alanine aminotransferase activity (U/L)	<109.5 (ref) ≥109.5	15.9 (13/82) 33.3 (15/45)	84.1 (69/82) 66.7 (30/45)	.03	2.7	1.1	6.3
Chloride (mmol/L)	≤106 (ref) 106 < Cl^-^ < 110 110 < Cl^-^ < 113 113 < Cl^-^ < 124	15.9 (7/44) 31.4 (11/35) 5.0 (1/20) 39.1 (9/23)	84.1 (37/44) 68.6 (24/35) 95.0 (19/20) 60.9 (14/23)	.04 .1 .24 .04	2.4 .3 3.4	.8 .03 1.1	1.7 2.4 10.9
Total bilirubin (μmol/L)	<7.5 (ref) ≥7.5	10.4 (5/48) 32.7 (18/55)	49.6 (43/48) 67.3 (37/55)	**.01**	4.2	1.4	12.4
**Others**							
Blood culture	Negative (ref) Positive	13.0 (12/92) 44.7 (17/38)	89.1 (82/92) 55.3 (21/38)	**<.001**	5.14	2.2	13.0

Boldface indicates statistically significant results (*P* < .05). Abbreviations: A/G = albumin/globulin; Cl- = chloride; HR = heart rate; OR = odds ratio; ref = reference; 95% CI = 95% confidence interval.

**Figure 1:**
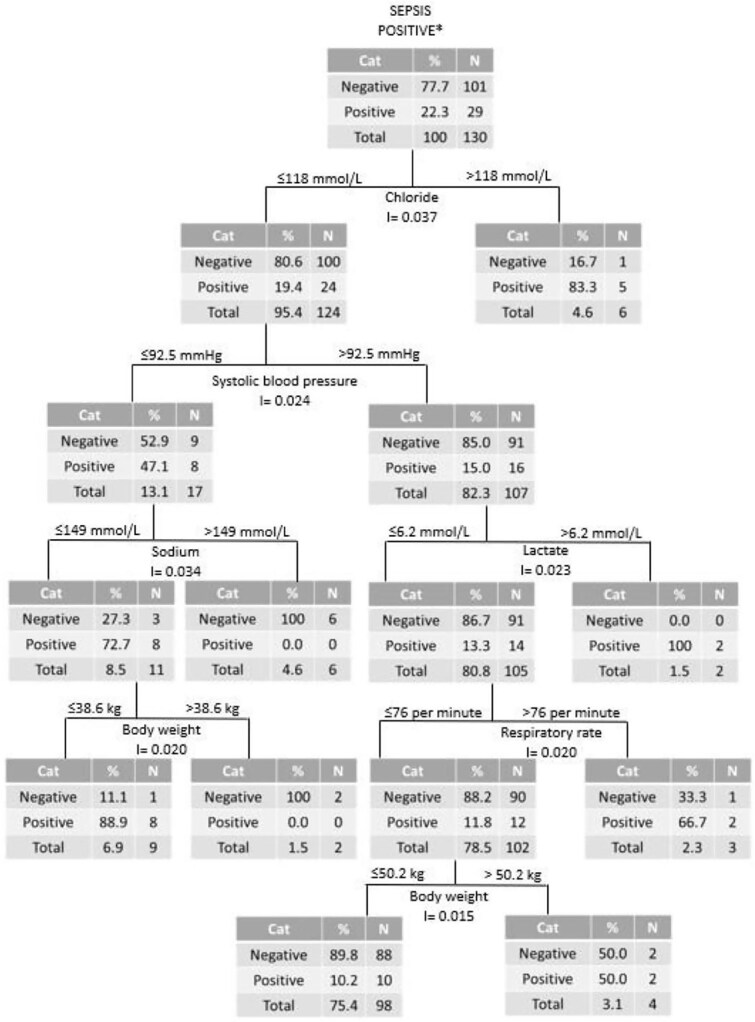
Decision tree for sepsis in 130 critically ill dogs based on clinical and laboratory variables with a sensitivity of 58.6%, a specificity of 97.0%, and an accuracy of 88.5%. *based on the proposed definition of sepsis. Abbreviations: Cat, category; *N*, number of dogs; I, improvement.

The multivariable models are described in Table 4. The clinical model (dyspnea, RR, and SBP) had a sensitivity of 42.9%, specificity of 92.1%, and accuracy of 79.8%. The combined model (dyspnea, RR, and alanine aminotransferase activity [ALT]) had a sensitivity of 35.0%, specificity of 92.8%, and accuracy of 79.8%. Decision tree analysis (Figure 1) indicated a sensitivity of 58.6% and specificity of 97.0%, with an accuracy of 88.5%. Respiratory rate (excluding panting) and SBP emerged as significant risk factors in both the multivariable and decision tree analyses.

**Table 4:** Results of multivariable logistic regression analysis for the association of clinical and laboratory variables with sepsis in 130 critically ill dogs.

**Variable **		**Category**	***P*-value**	**OR**	**95% CI**	**Se% (95% CI)**	**Sp%** **(95% CI)**	**Acc (%)**	**LR+**	**LR-**
**Clinical model**										
**Model A** ***n* = 84**	Dyspnea	Absent (ref) Present	.02	5.7	1.4-23.8	42.9 (21.7-64.0)	92.1 (85.4-98.7)	79.8	5.4	.6
	Respiratory rate	<38 (ref) ≥38	.02	4.8	1.3-17.4					
	Systolic blood pressure	≥90 mm Hg (ref) <90 mm Hg	.02	6.2	1.3-28.9					
**Combined model (clinical and laboratory variables)**										
**Model B** ***n* = 89**	Dyspnea	Absent (ref) Present	.008	6.5	1.6-26.0	35.0 (14.1-55.9)	92.8 (86.6-98.9)	79.8	4.9	.7
	Respiratory rate	<38 (ref) ≥38	.03	3.9	1.1-13.1					
	Alanine aminotransferase activity	<109.5 U/L (ref) ≥109.5 U/L	.03	3.8	1.1-12.8					
				

Boldface indicates statistically significant results (*P* < .05). Abbreviations: Acc = accuracy; LR+ = positive likelihood ratio; LR- = negative likelihood ratio; *N* = number of dogs; OR = odds ratio; ref = reference; Se = sensitivity; Sp = specificity; 95% CI = 95% confidence interval.

### Sepsis and critical illness scoring systems

Assessment of the currently used scoring systems in their association with sepsis or mortality is shown in Tables 5 and 6, respectively. Only the APPLE FAST score was associated with sepsis and with mortality.

**Table 5:** Diagnostic accuracy of available scoring systems for sepsis from human and canine medicine in a population of 130 critically ill dogs (2021-2024).

**Scoring systems**	**Categories**	**Septic dogs% (*N*)** **^a^**	***P*-value**	**OR**	**95% CI**		**Se (%)**	**Sp (%)**	**Acc (%)**	**LR+**	**LR-**
Original SIRS	Absent (ref) Present	6.7 (1/15) 24.3 (28/115)	.2	4.5	.6	35.8	96.6	13.9	32.3	1.1	.2
Modified SIRS2	Absent (ref) Present	10.8 (4/37) 26.9 (25/93)	.06	3.0	1.0	9.4	86.2	32.7	44.6	1.3	.4
Modified SIRS3	Absent (ref) Present	20.4 (19/93) 27.0 (10/37)	.4	1.4	.6	3.5	34.5	73.3	64.6	1.3	.9
APPLE FAST	<24.5 (ref) ≥24.5	16.4 (10/61) 36.2 (17/47)	.02	2.9	1.2	7.1	63.0	63.0	52.3	1.7	.6
Original qSOFA	0 (ref) 1 2 3	.0 (0/4) 21.9 (7/32) 19.1 (13/68) 36.0 (9/25)	.2 .8 .8 .2	.8 .8 2.5	.2 .2 .5	3.8 3.4 11.8	65.5	41.6	46.9	1.1	.8
Modified qSOFA	0 (ref) 1 2 3	20.0 (3/15) 14.9 (10/67) 29.7 (11/37) 45.5 (5/11)	.1 .6 .5 .2	.7 1.7 3.3	.2 .4 .6	2.9 7.2 18.9	55.2	68.3	65.4	1.7	.7
L2qSOFA	0 (ref) 1 2 3 4	.0 (0/3) 33.3 (4/12) 22.0 (9/41) 22.2 (10/45) 35.7 (5/14)	.8 1.0 1.0 1.0 1.0	/	/	/	85.7	12.6	30.4	1.0	1.1
L3qSOFA	0 (ref) 1 2 3 4 5	.0 (0/3) 29.4 (5/17) 20.9 (9/43) 27.3 (6/22) 21.1 (4/19) 36.4 (4/11)	.9 1.0 1.0 1.0 1.0 1.0	/	/	/	82.1	17.2	33.0	1.0	1.0
L4qSOFA	0 (ref) 1 2 3 4	.0 (0/3) 26.1 (6/23) 22.2 (12/54) 25.9 (7/27) 37.5 (3/8)	.9 1.0 1.0 1.0 1.0	/	/	/	78.6	23.0	36.5	1.0	.9
L5qSOFA	0 (ref) 1 2 3 4	.0 (0/3) 26.9 (7/26) 21.1 (12/57) 30.8 (8/26) 33.3 (1/3)	.9 1.0 1.0 1.0 1.0	/	/	/	75.0	25.3	37.4	1.0	1.0

Abbreviations: 95% CI = 95% confidence interval; APPLE FAST = acute patient physiologic and laboratory evaluation FAST; LqSOFA = qSOFA in combination with lactate; LR+ = positive likelihood ratio; LR- = negative likelihood ratio; OR = odds ratio; qSOFA = quick sequential organ failure assessment; ref = reference; Se = sensitivity; SIRS = systemic inflammatory response syndrome; Sp = specificity.

a Results are presented as x/y, where x indicates the number of critically ill dogs that were septic, and y displays the total number of critically ill dogs classified negative (absent) or positive (present) for the scoring system.

**Table 6:** Prognostic accuracy of available scoring systems for sepsis from human and canine medicine in a population of 130 critically ill dogs (2021-2024).

**Scoring systems**	**Categories**	**Observed death% (N)** **^a^**	***P*-value**	**OR**	**95% CI**		**Se (%)**	**Sp (%)**	**Acc (%)**	**LR+**	**LR-**
Original SIRS	Absent (ref) Present	20.0 (3/15) 39.1 (45/115)	.2	2.6	.7	9.6	93.8	14.6	43.8	1.1	.4
Modified SIRS2	Absent (ref) Present	32.4 (12/37) 38.7 (36/93)	.5	1.3	.6	2.9	75.0	30.5	46.9	1.1	.8
Modified SIRS3	Absent (ref) Present	35.5 (33/93) 40.5 (15/37)	.6	1.2	.6	2.7	31.3	73.2	57.7	1.2	.9
APPLE FAST	<24.5 (ref) ≥24.5	24.6 (15/61) 51.1 (24/47)	.01	3.2	1.4	7.2	65.9	66.7	53.8	2.0	.5
Original qSOFA	0 (ref) 1 2 3	28.6 (4/14) 26.3 (10/38) 37.5 (21/56) 59.1 (13/22)	.09 .9 .5 .08	.9 1.5 3.6	.2 .4 .9	3.5 5.4 15.2	27.1	89.0	66.2	2.5	.8
Modified qSOFA	0 (ref) 1 2 3	26.7 (4/15) 31.3 (21/67) 45.9 (17/37) 54.5 (6/11)	.2 .7 .2 .2	1.3 2.3 3.3	.4 .6 .6	4.4 8.7 17.2	12.5	93.9	63.8	2.0	.9
L2qSOFA	0 (ref) 1 2 3 4	33.3 (1/3) 25.0 (3/12) 22.0 (9/41) 46.7 (21/45) 50.0 (7/14)	.1 .8 .7 .7 .6	.7 .6 1.8 2.0	.04 .05 .1 .1	10.3 6.9 20.7 27.4	17.1	90.5	64.3	1.8	.9
L3qSOFA	0 (ref) 1 2 3 4 5	33.3 (1/3) 29.4 (5/17) 25.6 (11/43) 40.9 (9/22) 47.4 (9/19) 54.5 (6/11)	.4 .9 .8 .8 .7 .5	.8 .7 1.4 1.8 2.4	.06 .06 .1 .1 .2	11.4 8.3 17.7 23.4 34.9	14.6	93.2	65.2	2.1	.9
L4qSOFA	0 (ref) 1 2 3 4	33.3 (1/3) 26.1 (6/23) 27.8 (15/54) 51.9 (14/27) 62.5 (5/8)	.1 .8 .8 .6 .4	.7 .8 2.2 3.3	.05 .07 .2 .2	9.3 9.1 26.7 54.5	46.3	78.4	67.0	2.1	.7
L5qSOFA	0 (ref) 1 2 3 4	33.3 (1/3) 26.9 (7/26) 31.6 (18/57) 50.0 (13/26) 66.6 (2/3)	.3 .8 .9 .6 .4	.7 .9 2.0 4.0	.06 .08 .2 .1	9.5 10.9 24.9 119.2	36.6	81.1	65.2	1.9	.8

Abbreviations: 95% CI = 95% confidence interval; APPLE FAST = acute patient physiologic and laboratory evaluation FAST; LqSOFA = qSOFA in combination with lactate; LR+ = positive likelihood ratio; LR- = negative likelihood ratio; OR = odds ratio; qSOFA = quick sequential organ failure assessment; ref = reference; Se = sensitivity; SIRS = systemic inflammatory response syndrome; Sp = specificity.

a Results are presented as x/y, where x indicates the number of critically ill dogs that died, and y displays the total number of critically ill dogs classified negative (absent) or positive (present) for the scoring system.

## Discussion

In the absence of information about risk factors for sepsis in critically ill dogs and the diagnostic and prognostic value of available sepsis and critical illness scoring systems, we aimed to determine clinical and laboratory variables as risk factors for sepsis in 130 critically ill dogs. Additionally, associations between sepsis and mortality with available scoring systems used in humans and dogs were evaluated.

The first objective was to determine diseases associated with sepsis in critically ill dogs. Pneumonia and AKI were the most frequent comorbidities in septic dogs, both significantly associated with sepsis, whereas AKI was significantly associated with mortality.^5^^,^^23^ Pneumonia is a well-established cause of sepsis, with one-third of cases progressing to severe sepsis in both dogs and humans in intensive care unit (ICU) settings.^3^^,^^24^ A previous study^3^ focused on community-acquired pneumonia.^3^^,^^24^

Acute kidney injury is a common complication of critical illness and sepsis in dogs and humans, with high risk of mortality. In previous studies, AKI was considered as secondary organ damage to sepsis rather than a consequence of urosepsis.^3^^,^^25–28^ The odds of sepsis were 7.4 times higher in dogs with AKI than in dogs without AKI. Additionally, dogs with AKI had 5.0 times higher odds of mortality compared with dogs without AKI. In hospitalized septic dogs, the incidence of AKI has been reported to be 12.0%.^28^ However, outcomes may vary depending on availability and use of blood purification techniques.^29^^,^^30^ Although septic peritonitis was the third most common disease in combination with sepsis in our study, the association with sepsis was not significant, despite being frequently mentioned as a common cause in different studies.^31–33^ Other conditions that frequently occurred in combination with sepsis in our study included trauma, pyometra, intoxication, bacterial cystitis, and endocarditis. All trauma cases were bite injuries. Among these, trauma, pyometra, bacterial cystitis, and endocarditis previously have been identified as potential causes of sepsis.^32–36^ Because of the relatively limited number of observations of these conditions, conclusions regarding their association could not be established. Notably, comorbidities often were present (eg, pancreatitis combined with AKI) in our study cohort.

Sepsis was a common syndrome in critically ill dogs admitted to the emergency department of the veterinary teaching hospital (22.3%, 29/130) with high mortality rates (62.1%, 18/29). Septic dogs had a 3.9-fold higher risk of mortality compared with non-septic critically ill dogs. This finding emphasizes the importance of early and appropriate treatment of sepsis. These findings align with previously reported mortality rates (32.0%-80.0%).^5^^,^^37^^,^^38^ Most puppies were excluded because of insufficient body weight to obtain the required blood samples for the study. Nevertheless, sepsis remains a major cause of mortality in dogs within the first 3 weeks of life, probably related to the immunocompromised state of the neonate.^39^

Diagnosing sepsis in dogs remains challenging.^23^ Hence, our secondary aim was to identify clinical and laboratory variables associated with sepsis in critically ill dogs. Tachypnea was a significant predictor of sepsis, in both multivariable logistic regression (≥38 per minute) and CART analyses (≥76 per minute). This finding contrasts with previous research that did not observe this association, likely because of different sepsis definitions.^40^ Dyspnea was strongly associated with sepsis in both regression models but lacks comprehensive research in human and veterinary medicine. To our knowledge, only 1 study has identified dyspnea as a clinical sign in septic neonatal dogs, reporting its presence in 19.4% of the individuals.^39^ Hypotension (SBP <90 mm Hg) was significantly associated with sepsis. Similarly, a previous study found lower SBP (threshold <100 mm Hg) in septic dogs defined as SIRS with documented or suspected infection.^5^ Laboratory variables are also indicators for disease assessment, providing valuable insight into physiological imbalances and disease progression. Alanine aminotransferase, a marker of hepatocellular damage, was significantly associated with sepsis in multivariable analysis, consistent with studies in humans identifying increased ALT as a predictor of sepsis-related liver dysfunction.^41^^,^^42^ To the best of our knowledge, an association has not been described previously for sepsis in critically ill dogs. Chloride is essential for acid–base balance and for other physiological functions. Hyperchloremia can cause acidosis, which has been associated with increased mortality in humans and dogs.^43^^,^^44^ Chloride administration has been shown to adversely affect renal function in critically ill patients via vasoconstriction, decreased glomerular filtration rate, and decreased renal cortical perfusion.^43^^,^^45^^,^^46^ In our decision tree analysis, increased serum chloride concentrations were associated with sepsis. Sodium imbalance has been associated with increased mortality in critically ill humans and dogs, often because of disturbed water homeostasis.^43^ In our decision tree analysis, hyponatremia (≤149.0 mmol/L) exhibited increased odds of sepsis. Although sodium and chloride are physiologically correlated, decision trees do not assume independence between predictors. At each node, the model simply selects the variable that produces the largest reduction in impurity and so correlated variables only appear if each adds additional discriminatory value after the previous split. To our knowledge, no studies are available on the association of hyponatremia in septic humans or dogs. However, one study reported that hypo- (<145.0 mmol/L) and hyper-natremia (>154.0 mmol/L) occur in dogs with sepsis, with hyponatremia being associated with increased mortality rates in sick dogs.^47^ Lactate is a well-established biomarker of impaired tissue oxygenation, primarily attributable to decreased clearance, in humans and animals. Presence and severity of hyperlactatemia on admission have been recognized as predictors of mortality in critically ill humans and animals.^5^^,^^6^ Serial measurements are routinely employed for prognosis and treatment guidance.^5^^,^^6^^,^^48^ Surprisingly, in our study, increased lactate was associated with sepsis in only 2 dogs in CART. This finding could reflect the influence of other, more dominant, clinical predictors in the model. Because variable selection was based on univariable screening followed by stepwise backward elimination, the modeling approach was largely data-driven. As a result, some retained variables may reflect overlapping aspects of disease severity rather than distinct biological mechanisms (eg, tachypnea and dyspnea). Although conceptual collinearity was evaluated to ensure biological interpretability, biologically guided models excluding overlapping variables did not remain statistically significant. Therefore, the final models should be interpreted as exploratory, rather than as identifying independent causal predictors of sepsis. Our third objective was to determine the association of existing sepsis and critical illness scoring systems used in humans and dogs with sepsis or mortality in critically ill dogs. Screening tests should prioritize high sensitivity to minimize missed diagnoses (false-negative results), whereas confirmation tests should emphasize specificity to decrease unnecessary antimicrobial use (false-positive results).^49^ Considering diagnosis, only the APPLE FAST score was associated with sepsis, indicating its potential utility in predicting sepsis in critically ill dogs. The APPLE FAST score also exhibited the most balanced diagnostic performance in predicting sepsis, with a sensitivity and specificity of 63.0%. Nevertheless, the APPLE FAST score showed limited discriminatory power for ruling in or ruling out sepsis, as reflected by the likelihood ratios. One study reported that the APPLE FAST score could differentiate sepsis severity, as indicated by its association with both septic shock and the development of multiple organ dysfunction.^50^ In contrast, the original SIRS score demonstrated the highest sensitivity (96.6%), but it was not significantly associated with sepsis, confirming its limited predictive value as a single diagnostic tool, as previously reported.^1^^,^^8^ Regarding mortality, only the APPLE FAST score was associated with mortality in critically ill dogs, consistent with previous research in dogs, and it had moderate sensitivity (65.9%) and specificity (66.7%). This association reflects its ability to identify dogs with higher degrees of organ dysfunction.^28^^,^^33^^,^^35^ In our study, the original qSOFA score was not associated with mortality and exhibited a low sensitivity (27.1%) but high specificity (89.0%). These findings are consistent with those of a previous study,^4^ in a similar population of critically ill dogs.^4^^,^^5^ However, this observation contrasts with other studies, which did find an association between an original qSOFA score of ≥2 and increased odds of mortality.^7^^,^^12^ The original qSOFA score was developed to assess the severity of organ dysfunction and predict whether or not hyperlactatemia (>2.0 mmol/L) could serve as an additional variable to the original qSOFA score in identifying humans with increased mortality risk.^6^ However, adding lactate to the original qSOFA score did not improve its predictive performance, because its sensitivity remained low.^51^ Notably, likelihood ratios indicated limited ability of scoring systems to rule in or rule out mortality.

The original SIRS criteria had the highest sensitivity (93.8%) in predicting mortality in our population of critically ill dogs. In a triage setting, where early identification of at-risk patients is critical, a scoring system with high sensitivity is particularly valuable. Physiological differences between humans and dogs may limit direct extrapolation of scoring systems. For example, the qSOFA RR threshold (≥22 per minute) is lower than physiological values in dogs (>40 per minute). Additionally, panting is often present in non-pathological situations, which further complicates interpretation of RR in dogs and subsequently scoring system results.^5^^,^^7^ Adjusting RR threshold to >40 per minute to reflect physiology in dogs increased specificity but decreased sensitivity in predicting mortality, raising concerns about potential delays in treatment initiation, which could adversely affect clinical outcomes. A similar finding was reported in a previous study on dogs with severe sepsis and septic shock.^33^ Scoring systems should not be used as a standalone tool to guide decision-making, as advised by the Surviving Sepsis Campaign.^4^ Given that screening tests prioritize high sensitivity, the moderate sensitivity of the APPLE FAST score for diagnostic and prognostic purposes is too low to be considered adequate for screening use.

Our study had some limitations. First, power analysis was not performed, potentially resulting in an insufficient sample size and a decreased ability to detect certain associations (eg, septic peritonitis and sepsis). Second, the absence of a reference standard for sepsis diagnosis in dogs may have led to misclassification bias. Third, the proposed definition of sepsis relied on strict criteria, which may have led to misclassification of some septic dogs into the non-septic critically ill group. However, this definition was based on the Sepsis-3 definition used in humans, which similarly incorporates organ dysfunction as a central component. Additionally, applying less stringent criteria (SIRS criteria combined with positive blood culture), would have resulted in 36 dogs being categorized as septic instead of 29. These less strict criteria increase the risk of misclassifying bacteremic dogs as septic. Fourth, because of the low specificity of the SIRS criteria, the critically ill non-septic group likely represented a biologically heterogeneous population. Consequently, comparisons between septic and non-septic dogs should be interpreted as risk stratification within a SIRS-positive cohort rather than as a contrast between true disease and a non-diseased (healthy) control group. Fifth, blood gas analysis was not routinely performed to assess the clinical relevance of hyperchloremia. Sixth, AKI staging, particularly for grade I, remains challenging. Because doing so was beyond the scope of our study, differentiation between grade I and grade II was not performed. Finally, external validation was not performed. Also, some of our predictors were related to our sepsis definition, which could have led to incorporation bias. Hence, results of our study should be considered as exploratory regarding risk factors for sepsis and as predictive (evaluation) for available scoring systems. Therefore, extrapolation of the results should be approached with caution.

In conclusion, sepsis is common in critically ill dogs and is associated with increased mortality risk. Pneumonia and AKI were key comorbidities. Risk factors such as tachypnea, dyspnea, hypotension, body weight, increased ALT activity, hyperchloremia, hyponatremia, and hyperlactatemia could guide in the early recognition of sepsis. Additionally, these variables could be valuable to integrate into clinical scoring systems. Based on the population of dogs and the clinical setting of our study, the APPLE FAST score showed an association with sepsis and mortality. However, its moderate sensitivity limits its applicability as a screening tool for both sepsis and mortality.

## Supplementary Material

Supplementary_materials_aalag168

## Off-label antimicrobial declaration

Authors declare no off-label use of antimicrobials.

## Institutional animal care and use committee or other approval declaration

Authors declare no institutional animal care and use committee or other approval was needed.

## Abbreviations

AHDS: acute hemorrhagic diarrhea syndrome
AKI: acute kidney injury
ALT: alanine aminotransferase activity
APPLE FAST: acute patient physiologic and laboratory evaluation
FAST, BG: blood glucose
BPM: beats per minute
BUN: blood urea nitrogen
CART: classification and regression tree
CAT: category
CI: confidence interval
CRT: capillary refill time
DIC: disseminated intravascular coagulation
DKA: diabetic ketoacidosis
FUO: fever of unknown origin
G: gauge
GDV: gastric dilatation volvulus
HPF: high-power field
HR: heart rate
I: improvement
ICU: intensive care unit
IRIS: International Renal Interest Society
IV: intravenous
LR+: positive likelihood ratio
LR-: negative likelihood ratio
L2: lactate 2
LqSOFA qSOFA: in combination with lactate
MALDI-TOF MS: matrix-assisted laser desorption/ionization time-of-flight mass spectrometry
Max: maximum
Min: minimum
OR: odds ratio
POCUS: point-of-care ultrasonography
qSOFA: quick sequential organ failure assessment
Ref: reference
ROC: receiver operating characteristic
RR: respiratory rate
SBP: systolic blood pressure
SD: standard deviation
Se: sensitivity
SIRS: systemic inflammatory response syndrome
Sp: specificity
STROBE: strengthening the reporting of observational studies in epidemiology
TP: total protein
UPC: urine protein/creatinine ratio
