# Supplementary material for: Sepsis in critically ill dogs: identification of clinical and laboratory risk factors and evaluation of sepsis scoring systems
Source: J Vet Intern Med. 2026 Aug 13;40(4):aalag168. doi: 10.1093/jvimsj/aalag168 (PMC13471268; doi:10.1093/jvimsj/aalag168)
Supplement: Supplementary file 1 [file supplementary_materials_aalag168.docx]

| *Supplementary Table 1. Overview of the evaluated scoring systems* | | | | | | | |
| --- | --- | --- | --- | --- | --- | --- | --- |
|  | **Original SIRS**^7^ | **Modified SIRS2**^7^ | **Modified SIRS3**^7^ | **Original qSOFA**^5,12^ | **Modified qSOFA** | **LqSOFA**^5^ | **APPLE FAST**^11^ |
| **Criteria** | ≥2 criteria | ≥2 criteria | ≥3 criteria | One point of criterion was met, total score between 0-3 | One point of criterion was met, total score between 0-3 | One point of criterion was met, total score between 0-3 | See Supplementary Figure 1 |
| **Respiratory rate (per minute)** | >20 (excl. panting) | >40 (excl. panting) | >40 (excl. panting) | ≥22 (excl. panting) | >40 (excl. panting) | ≥22 (excl. panting) |  |
| **Heart rate (beats per minute)** | >120 | >150 | >150 | / | / | / |  |
| **Temperature (°C)** | <38.1 or >39.2 | <38.1 or >39.2 | <38.1 or >39.2 | / | / | / |  |
| **Mentation** | / | / | / | Altered | Altered | Altered |  |
| **Systolic blood pressure (mmHg)** | / | / | / | ≤100 | ≤100 | ≤100 |  |
| **Leukocyte count (x10^9^/L)** | <6.0 or >16.6 | <6.0 or >16.6 | <6.0 or >16.6 | / | / | / |  |
| **Lactate (mmol/L)** | / | / | / | / | / | Add 1 point if lactate >2.0 (L2qSOFA), >3.0 (L3qSOFA), >4.0 (L4qSOFA), or >5.0 (L5qSOFA).  No change if ≤2.0 |  |

| *Supplementary Table 2. Non-significant results of univariable logistic regression analysis for the association of clinical and laboratory variables with sepsis in 130 critically ill dogs* | | | | | | | |
| --- | --- | --- | --- | --- | --- | --- | --- |
| **Variable** | **Categories** | **Septic dogs% (number of septic dogs/number of dogs in the category) or (median and min-max)** | **Non-septic CI dogs % (number of septic dogs/number of dogs in the category) or (median and min-max)** | ***p*-value** | **OR** | **95% CI** | |
| **Signalment** | | | | | | | |
| Size | Small <15 kg (ref)  Medium 15-25 kg  Large >25 kg | 23.7 (9/38)  17.6 (6/34)  23.2 (13/56) | 76.3 (29/38)  82.4 (28/34)  76.8 (43/56) | .79  .53  .96 | .7  1.0 | .2  .4 | 2.2  2.6 |
| Age (days) | <1520 (ref)  ≥1520 | 18.4 (7/38)  23.9 (22/92) | 81.6 (31/38)  76.1 (70/92) | .5 | 1.4 | .5 | 3.6 |
| Sex | Male (ref)  Female | 21.1 (15/71)  23.7 (14/59) | 78.9 (56/71)  76.3 (45/59) | .7 | 1.2 | .5 | 2.7 |
| Body weight (kg) | <30.3 (ref)  ≥30.3 | 18.7 (17/91)  29.7 (11/37) | 81.3 (74/91)  70.3 (26/37) | .2 | 1.8 | .8 | 4.4 |
| **Clinical variables** | | | | | | | |
| Mental status | Alert (ref)  Lethargy  Apathy  Coma | 19.0 (4/21)  13.5 (7/52)  31.5 (17/54)  50.0 (1/2) | 81.0 (17/21)  86.5 (45/52)  68.5 (37/54)  50.0 (1/2) | .13  .55  .29  .34 | .7  2.0  4.3 | .2  .6  .2 | 2.5  6.7  83.5 |
| Respiratory rate (per minute)  (excluding panting in n=42) | Continuous | Median: 44.0  Min-Max, 16.0-104.0 | Median: 36.0  Min-Max, 16.0-80.0 | .01 | 1.0 | 1.0 | 1.1 |
| Lung auscultation | Normal (ref)  Abnormal | 8.8 (8/91)  28.9 (11/38) | 91.2 (83/91)  71.1 (27/38) | .3 | 1.7 | .7 | 4.0 |
| Heart rate (beats per minute) | <131 (ref)  ≥131 | 17.5 (10/57)  25.0 (18/72) | 82.5 (47/57)  75.0 (54/72) | .3 | 1.6 | .7 | 3.7 |
| Heart auscultation | Normal (ref)  Abnormal | 13.5 (5/37)  25.8 (24/93) | 86.5 (32/37)  74.2 (69/93) | .1 | 2.2 | .8 | 6.4 |
| Mucosal membrane color (abnormal) | Normal/ pink (ref)  Abnormal | 16.7 (8/48)  25.6 (21/82) | 83.3 (40/48)  74.4 (61/82) | .2 | 1.7 | .7 | 4.3 |
| Capillary refill time (seconds) | ≤2 (ref)  <1  >2 | 17.4 (12/69)  21.6 (8/37)  33.3 (7/21) | 82.6 (57/69)  78.4 (29/37)  66.6 (14/21) | .3  .6  .1 | 1.3  2.4 | .5  .8 | 3.6  7.1 |
| Lymph nodes | Normal (ref)  Enlarged | 24.3 (27/111)  10.5 (2/19) | 75.7 (84/111)  89.5 (17/19) | .2 | .4 | .08 | 1.7 |
| Pulse quality | Normal (ref)  Strong  Weak | 14.0 (6/43)  13.3 (2/15)  26.3 (5/19) | 86.0 (37/43)  86.7 (13/15)  73.7 (14/19) | .5  1.0  .2 | .9  2.2 | .2  .6 | 5.3  8.4 |
| Temperature (°C) | <39.2 (ref)  ≥39.2 | 14.5 (9/62)  29.4 (20/68) | 85.5 (53/62)  70.6 (48/68) | .05 | 2.5 | 1.0 | 5.9 |
| Skin turgor (seconds) | ≤2 (ref)  >2 | 22.3 (25/112)  23.5 (4/17) | 77.7 (87/112)  76.5 (13/17) | .9 | 1.1 | .3 | 3.6 |
| Body condition score | Ideal weight (4-6, ref)  Underweight (1-3)  Overweight (7-9) | 23.5 (24/102)  18.8 (3/16)  12.5 (1/8) | 76.5 (78/102)  81.3 (13/16)  87.5 (7/8) | .7  .7  .5 | .8  .5 | .2  .05 | 2.9  4.0 |
| Abdomen inspection | Normal (ref)  Distended | 22.1 (27/122)  33.3 (2/6) | 77.9 (95/122)  66.6 (4/6) | .5 | 1.8 | .3 | 10.1 |
| Abdominal palpation | Normal (ref)  Tense | 26.0 (20/77)  18.4 (9/49) | 74.0 (57/77)  81.6 (40/49) | .3 | .6 | .3 | 1.6 |
| Abdominal undulation (free fluids) | Negative (ref)  Positive | 23.5 (28/119)  11.1 (1/9) | 76.5 (91/119)  88.9 (8/9) | .4 | .4 | .05 | 3.4 |
| Lameness | Normal (ref)  Lame | 21.2 (22/104)  22.2 (4/18) | 78.8 (82/104)  77.8 (14/18) | .9 | 1.1 | .3 | 3.6 |
| Joints | Normal (ref)  Swollen | 22.3 (25/112)  23.1 (3/13) | 77.7 (87/112)  76.9 (10/13) | .1 | 1.0 | .3 | 4.1 |
| Vaginal discharge | Absent (ref)  Present | 23.2 (13/56)  25.0 (1/4) | 76.8 (43/56)  75.0 (3/4) | .9 | 1.1 | .1 | 11.5 |
| Wounds | Absent (ref)  Present | 19.2 (19/99)  33.3 (10/30) | 80.8 (80/99)  66.7 (20/30) | .1 | 2.1 | .8 | 5.2 |
| Urination abnormalities | Absent (ref)  Present | 20.2 (19/94)  28.6 (8/28) | 79.8 (75/94)  71.4 (20/28) | .4 | 1.6 | .6 | 4.1 |
| Dental health | Normal (ref)  Abnormal | 14.8 (9/61)  28.0 (14/50) | 85.2 (52/61)  72.0 (36/50) | .09 | 2.2 | .9 | 5.7 |
| Systolic blood pressure (mmHg) | ≥90 (ref)  <90 | 21.0 (22/105)  46.2 (6/13) | 79.0 (83/105)  53.8 (7/13) | .05 | 3.2 | 1.0 | 10.6 |
| Antimicrobial use before sampling/admission | No (ref)  Yes | 25.8 (16/62)  20.6 (13/63) | 74.2 (46/62)  79.4 (50/63) | .5 | .7 | .3 | 1.7 |
| **Complete blood count** | | | | | | | |
| White blood cells (10^9^/L) | <15.2 (ref)  ≥15.2 | 18.2 (14/77)  29.4 (15/51) | 81.8 (63/77)  70.6 (36/51) | .1 | 1.9 | .8 | 4.3 |
| Neutrophils (10^9^/L) | <23.2 (ref)  ≥23.2 | 21.0 (22/105)  31.8 (7/22) | 79.0 (83/105)  68.2 (15/22) | .3 | 1.8 | .6 | 4.8 |
| Basophils (10^9^/L) | .0-.1 (ref)  >.1 | 23.1 (27/117)  20.0 (2/10) | 76.9 (90/117)  80.0 (8/10) | .8 | .8 | .2 | 4.2 |
| Eosinophils (10^9^/L) | .06-1.2 (ref)  <.06  >1.2 | 18.9 (10/53)  26.4 (19/72)  .0 (0/2) | 81.1 (43/53)  73.6 (53/72)  100 (2/2) | .6  .3  1.0 | 1.5  0-0.0 | .6  .0 | 3.7  / |
| Thrombocytes (10^9^/L) | <70.5 (ref)  ≥70.5 | 11.8 (2/17)  24.5 (27/110) | 88.2 (15/17)  75.5 (83/110) | .3 | 2.4 | .5 | 11.4 |
| Hematocrit (%) | <39.4 (ref)  ≥39.4 | 20.0 (10/50)  24.7 (19/77) | 80.0 (40/50)  75.3 (58/77) | .5 | 1.3 | .6 | 3.1 |
| Red blood cells (10^12^/L) | <6.1 (ref)  ≥6.1 | 18.6 (8/43)  25.0 (21/84) | 81.4 (35/43)  75.0 (63/84) | .4 | 1.5 | .6 | 3.6 |
| Reticulocytes (K/µL) | continuous | Median: 35.3  Min-Max, 13.5-142.7 | Median: 32.7  Min-Max, .6-434.7 | .3 | 1.0 | 1.0 | 1.0 |
| **Biochemistry** | | | | | | | |
| Albumin (g/L) | <19.5 (ref)  ≥19.5 | 14.3 (2/14)  23.9 (27/113) | 85.7 (12/14)  76.1 (86/113) | .4 | 1.9 | .4 | 8.9 |
| Alkaline phosphatase (U/L) | <104.5 (ref)  ≥104.5 | 5.3 (1/19)  25.5 (28/110) | 94.7 (18/19)  74.5 (82/110) | .08 | 6.1 | .8 | 48.2 |
| Blood urea nitrogen (mmol/L) | <29.9 (ref)  ≥29.9 | 21.1 (24/114)  45.5 (5/11) | 78.9 (90/114)  54.5 (6/11) | .07 | 3.2 | .9 | 11.5 |
| BUN/Creatinine | Continuous | Median: 15.0  Min-Max, 5.0-96.3 | Median: 16.0  Min-Max, 4.0-90.0 | .4 | 1.0 | 1.0 | 1.0 |
| Creatinine (µmol/L) | <117.5 (ref)  ≥117.5 | 18.6 (18/97)  35.5 (11/31) | 81.4 (79/97)  64.5 (20/31) | .05 | 2.4 | 1.0 | 5.9 |
| Globulins (g/L) | <37.5 (ref)  ≥37.5 | 19.4 (18/93)  31.0 (9/29) | 80.6 (75/93)  69.0 (20/29) | .2 | 1.9 | .7 | 4.8 |
| Blood glucose (BG) (mmol/L) | ≤4.5 (ref)  4.5 < BG < 5.5  5.5 < BG < 6.3  6.3 < BG < 30.5 | 30.8 (12/39)  19.4 (7/36)  33.3 (6/18)  11.1 (4/36) | 69.2 (27/39)  80.6 (29/36)  66.7 (12/18)  88.9 (32/36) | .2  .3  .8  .05 | .5  1.1  .3 | .2  .3  .08 | 1.6  3.7  1.0 |
| Total protein (g/L) | <68.5 (ref)  ≥68.5 | 20.0 (20/100)  29.6 (8/27) | 80.0 (80/100)  70.4 (19/27) | .3 | 1.7 | .6 | 4.4 |
| Sodium (mmol/L) | <153.5 (ref)  ≥153.5 | 18.8 (19/101)  40.0 (8/20) | 81.2 (82/101)  60.0 (12/20) | .04 | 2.9 | 1.0 | 8.0 |
| Total protein (g/L) | ≤56.0  56.0 < TP < 61.0  61.0 < TP < 67.0  67.0 < TP < 86.0 | 29.3 (12/41)  20.0 (6/30)  7.7 (2/26)  26.7 (8/30) | 70.7 (29/41)  80.0 (24/30)  92.3 (24/26)  73.3 (22/30) | .2  .4  .05  .8 | .6  .2  .9 | .2  .04  .3 | 1.9  1.0  2.5 |
| Potassium (mmol/L) | <4.5 (ref)  ≥4.5 | 23.9 (17/71)  21.6 (11/51) | 76.1 (54/71)  78.4 (40/51) | .8 | .9 | .4 | 2.1 |
| Lactate (mmol/L) | <1.7 (ref)  ≥1.7 | 18.4 (7/38)  27.3 (21/77) | 81.6 (31/38)  72.7 (56/77) | .3 | 1.7 | .6 | 4.3 |
| **Abbreviations**: OR, odds ratio; 95% CI, 95% confidence interval; ref, reference; min, minimum; max, maximum; BUN, blood urea nitrogen; BG, blood glucose; TP, total protein | | | | | | | |

Supplementary Figure 1. Criteria used for the APPLE FAST score. The mentation score was defined as follows: 0=normal mentation; 1=able to stand unassisted, responsive but dull; 2=able to stand only with assistance, responsive but dull; 3=unable to stand, responsive; and 4=unable to stand, unresponsive.^11^
